# ICESsuHN105, a Novel Multiple Antibiotic Resistant ICE in *Streptococcus suis* Serotype 5 Strain HN105

**DOI:** 10.3389/fmicb.2019.00274

**Published:** 2019-02-26

**Authors:** Yinchu Zhu, Yue Zhang, Jiale Ma, Wenyang Dong, Xiaojun Zhong, Zihao Pan, Huochun Yao

**Affiliations:** ^1^College of Veterinary Medicine, Nanjing Agricultural University, Nanjing, China; ^2^OIE Reference Lab for Swine Streptococcosis, Nanjing, China; ^3^Key Laboratory of Animal Bacteriology, Ministry of Agriculture, Nanjing Agricultural University, Nanjing, China

**Keywords:** *S. suis* serotype 5, comparative genomics, multi-antibiotic resistance, integrative and conjugative element, natural transformation, bacteriocin

## Abstract

*Streptococcus* *suis* serotype 5, an emerging zoonosis bacterial pathogen, has been isolated from infections in both pigs and humans. In this study, we sequenced the first complete genome of a virulent, multidrug-resistant SS5 strain HN105. The strain HN105 displayed enhanced pathogenicity in zebrafish and BABL/c mouse infection models. Comparative genome analysis identified a novel 80K integrative conjugative element (ICE), ICESsuHN105, as required for the multidrug resistance phenotype. Six corresponding antibiotic resistance genes in this ICE were identified, namely *tet* (O), *tet* (M), *erm* (two copies), *aph*, and *spc*. Phylogenetic analysis classified the element as a homolog of the ICESa2603 family, containing the typical family backbone and insertion DNA. DNA hybrids mediated by natural transformation between HN105 and ZY05719 verified the antibiotic resistant genes of ICESsuHN105 that could be transferred successfully, while they were dispersedly inserted with a single gene in different genomic locations of ZY05719_(HN105)_ transformants. To further identify the horizontal transfer of ICESsuHN105 as a whole mobile genetic element, a circular intermediate form of ICESsuHN105 was detected by PCR. However, the effective conjugation using serotype 2 *S. suis* as recipients was not observed in current assays *in vitro*. Further studies confirmed the presence of the complete lantibiotic locus encoded in ICESsuHN105 that effectively inhibits the growth of other streptococci. In summary, this study demonstrated the presence of antibiotic resistance genes in ICE that are able to transfer between different clinical isolates and adapt to a broader range of *Streptococcus* serotype or species.

## Introduction

*Streptococcus suis* is a major swine pathogen that causes severe diseases in pigs, inducing meningitis, septicemia, arthritis, and endocarditis (Gottschalk et al., 2010; Fulde and Valentin-Weigand, 2013). Furthermore, *S. suis* is a zoonotic human pathogen affecting those in close contact with infected pigs or pig products (Huong et al., 2014; Kerdsin et al., 2017). Several outbreaks in Southeast Asia highlighted its danger to public health (Yu et al., 2006). *S. suis* serotype 2 (SS2) is the most prevalent in human infections and is relatively well studied. However, little is known about other serotypes, such as *S. suis* serotype 5 (SS5), which have arisen over recent decades. There have been four reported cases of human SS5 infections since 2011 around the world, including a patient with spontaneous bacterial peritonitis in Thailand, a pig farmer with septic arthritis in Sweden, a pig farmer in United States with Streptococcal toxic shock syndrome and a patient with bacteremia in Japan (Kerdsin et al., 2011; Gomez et al., 2014; Gustavsson and Rasmussen, 2014; Yoshida et al., 2017). Despite the low number of reported human cases, studying the occurrence and transmission of SS5 is important in light of the severity of the infections and the danger of the rise in antibiotic resistance. The abuse of antibiotics in multiple fields, including aquaculture and the food industry, and the genetic variation of *S. suis* via acquiring and transferring of genes, has resulted in an unprecedented rise of the antibiotic resistance phenomenon (Soares et al., 2014). Thus, it is critically important to better understand the characteristics of *S. suis* from both pathogenic and antibiotic-resistance perspectives.

The two significant streptococcal horizontal gene transfer mechanisms are mobile genetic elements such as integrative and conjugative elements (ICEs), and the naturally occurring competence for DNA transformation. These properties enhance bacterial adaptation to environmental conditions promoting bacterial evolution due to the acquisition of virulence factors, antibiotic resistance genes, and/or toxin–antitoxin systems (Huang J. et al., 2016). ICEs carry genes required for various survival traits, are integrative into the host chromosomes for stable transmission, and are capable of excision, circularization, and transfer via conjugation. The natural transformation abilities could mediate genomic hybridization via homologous recombination, following uptake of foreign DNA from the environment.

At the time of this study, most of the complete genomes of *S. suis* available in the NCBI database were SS2 strains. In this study, we present a first complete genome sequence of a virulent, multidrug-resistant SS5 (genotype) strain, HN105. A comprehensive genomic analysis with two SS2 virulent strains was performed to characterize the HN105 genome. The result demonstrated that strain HN105 contains a novel ICE(80K), denoted ICESsuHN105. Furthermore, the deletion of this 80K genomic island confirmed the role that this ICE is required for multi-antibiotic resistance. In this study we also proved that the natural transformation for gene hybrids may show one way of horizontal gene transfer for antibiotic resistance genes between different serotype strains. Indeed, a lantibiotic gene cluster was identified in the ICE and was confirmed to confer to the donor strain a significant growth advantage in inter-bacterial competition.

## Materials and Methods

### Bacterial Strains, Culture Conditions, and DNA Extraction

The bacterial strains used in this study are listed in Table 1. The strain HN105 was isolated from the knee joint fluid of a diseased pig in Henan province, China in 2014. The SS2 virulent strain ZY05719 was isolated in 2005 from a pig with acute septicemia during an epidemic in Ziyang, China (Zhang and Lu, 2007). The reference virulent strain P1/7 was originally isolated from a diseased pig with meningitis (Wu et al., 2014b). All strains were grown in Todd-Hewitt broth (THB, BD) or on agar at 37°C with 5% CO_2_. Chloramphenicol (5 μg/ml) was used, when necessary. Total genomic DNA was extracted using an Omega Bacteria DNA Kit (OMEGA, China) according to the manufacturer’s instructions. All primers used in this study are listed in Supplementary Table S1.

**Table 1:** The information of sequenced strains used for genomic comparison.

Strains	Serotype	Size(M)	GC%	ST	GenBank accession number	Place of origin
ZY05719	2	1.99	41.1	7	CP007497.1	China
P1/7	2	1.91	41.2	1	NC_012925.1	Europe


### Virulence Assessment Using Zebrafish and Mouse Infection Models

Zebrafish and BABL/c mice infections were performed according to previous studies (Wu et al., 2014a; Dong et al., 2015). The *S. suis* strains were grown to mid-log phase (about OD_600_ = 0.6), washed three times with 1× PBS (pH 7.4), and diluted to different doses in PBS before injection. Eight BALB/c (5-week-old) mice or 15 zebrafish for each infection group were injected intraperitoneally with the bacterial suspensions and mortality was recorded for 7 days post-infection. The mice were injected with 5 × 10^8^ CFU and 2 × 10^8^ CFU, then monitored three times a day; loss of mobility was set as a predictor of death during observation. Zebrafish were injected with 1 × 10^5^ CFU, 1 × 10^6^ CFU, 1 × 10^7^ CFU, and 1 × 10^8^ CFU. The moribund mice, recovered mice, and the mice in the control group were anesthetized at the end of the 7-day observation period with pelltobarbitalum natricum (30 mg/kg), and sacrificed by cervical dislocation. The zebrafish received lethal anesthesia with 3-aminobenzoic acid ethyl ester methanesulfonate MS-222 (90 mg/L).

### Growth Curve of *S. suis* Strains

The overnight *S. suis* cultures in THB medium, grown to an OD_600_ of 0.6, were sub-cultured again into 50 ml THB at a 1:100 dilution. The optical densities at OD_600_ of bacterial cultures were measured periodically until the late stationary phase (15 h after seeding, 1 h intervals).

### Antibiotic Susceptibility Test

An antibiotic susceptibility test was performed by assessing the minimum inhibitory concentration (MIC) of strain HN105, P1/7 and ZY05719. Eighteen antibiotics were used, including some important antibiotics such as erythromycin, vancomycin, tetracycline, cephalosporins, chloramphenicol and kanamycin. The test was performed by the broth microdilution method, according to the standard methods in the Clinical and Laboratory Standards Institute (CLSI) guidelines (2016). Briefly, the overnight bacterial culture was diluted (1:1000). Equal volumes of the bacterial culture were added in the 96-well plates and mixed with serially diluted antibiotics. Following 16 h incubation at 37°C with CO_2_, the growth in each well was recorded. The highest dilution of the antibiotic that inhibited bacterial growth, compared to the media control, was recorded as the MIC.

### High-Throughput Genome Sequencing, Assembly, and Annotation

The whole genome sequencing of HN105 was performed on the Illumina Miseq and Pacbio RSII platforms (Personalbio Bio-Technology Co., Ltd.). Using SPAdes (version 3.7.1), and the second generation and third generation sequencing data were assembled. Using the MUMmer software for co-linearity analysis with the reference genome, the positional relationship of contigs on the genome was determined. The gaps between the contigs were repaired using the original data from the third-generation sequencing. Finally, the complete chromosomal sequence was corrected using Pilon software. For plasmids, the contigs were compared to the nucleotide library in the NCBI database, the plasmid sequence was picked out, and the third-generation sequencing data was used to fill the gaps as noted above in order to obtain a complete plasmid sequence. A total of 2,196,724 bp for chromosomal DNA were generated by the combination of the two libraries (PE400 and S10K). The open reading frames (ORFs) were identified using Glimmer 3.0 based on the HN105 genome sequence (Delcher et al., 1999). The tRNAs and rRNAs were predicted by tRNAscan-SE 1.31 and RNAmmer 1.2, respectively (Lowe and Eddy, 1997), whereas ncRNA was BLAST-analyzed to the Rfam database (Lagesen et al., 2007). The CRISPR recognition tool (CRT) was used to predict DRs (forward repeats) and spacers (Bland et al., 2007). The GIs of HN105 were determined with Island Viewer^1^. The sequences and annotations of HN105 were deposited with GenBank^2^ under accession no. CP029398 and no. CP029399 for the plasmid.

### Multi-Locus Sequence Typing (MLST)

By comparing the sequences of seven housekeeping genes (*aroA, cpn60, dpr, gki, mutS, recA*, and *thrA*) in *S. suis*, MLST provides a discriminatory genotyping method for different strains (Maiden et al., 2013). The allele numbers and sequence type (ST) of strains used in this study were downloaded from the MLST database^3^ and analyzed with eBURST^4^.

### Comparative Genomic Analyses of HN105

Strains P1/7 and ZY05719 were representative clinical and virulent SS2 strains, respectively (Table 1). A comparison of HN105 with the above SS2 strains were performed using the program progressiveMauve (Darling et al., 2010), displaying multiple genome alignments.

### Determination of the ICESsuHN105 Sequence

A comparison of HN105 with P1/7 and ZY05719 was performed using progressiveMauve (Darling et al., 2010) and a major genomic island discovered was determined to be an ICE element.

Annotation of the complete ICE sequence was conducted with RAST (Aziz et al., 2008) and the results were manually checked by comparison with the NCBI database^5^. Comparative analysis was carried out with the BLAST program^6^.

### Phylogenetic Analysis of the ICEs in *S. suis*

A recent study identified 30 core genes of ICEs in *S. suis*, primarily those encoding integrase, excisionase, relaxase, plasmid mobilization relaxosome protein, type IV secretion systems, DNA primase, replication initiator protein A, DNA 5′-methyltransferase and hypothetical proteins (Huang J. et al., 2016). Using this data, we compared the HN105 ICE genes with the corresponding genes of ICESa2603 using BLAST analysis and the ACT software in order to build phylogenetic trees. In all, 13 ICEs were used to generate the phylogenetic tree on MEGA6, using the maximum-likelihood method with bootstrapping.

### Construction of the *Δ80K* Mutant and Its Role in Antibiotic Resistance

The unique 80K genomic island was knocked out by natural transformation, according to a recent study (Zaccaria et al., 2014). The forward and reverse homologous sequences of the target gene were fused with the chloramphenicol marker by overlap PCR. Then the DNA products were mixed with the peptide, incubated, and selected on THB agar (Cm^R^). MICs were then determined for kanamycin, gentamicin, erythromycin, spectinomycin, tetracycline, lincomycin, and neomycin.

### Construction of ZY05719 Multi-Antibiotic Transformants

Genomic DNA was extracted from HN105 using the Omega genomic DNA purification kit (OMEGA, China). The transformants were generated by natural transformation of HN105 DNA into ZY05719 (SS2) on THB agar, as described previously (Zaccaria et al., 2014). The receptor strain ZY05719 was cultured in THB until OD about 0.04, and HN105 DNA with pheromones peptide were mixed with 100 μl THY bacteria. After 2 h of incubation at 37°C, samples were spread on plates (Kan^+^ and Spc^+^). Of note, HN105 is multidrug-resistant (spectinomycin, kanamycin, tetracycline and erythromycin) and conferred chromosomally, whereas ZY05719 is susceptible to all three. The antibiotic resistance of transformants was tested on THB agar with 100 μg/ml spectinomycin and 50 μg/ml kanamycin. The transformants were cultured and the DNA extracted for genome sequencing.

### Detection of Excision and Circularization of ICESsuHN105, and Mating Assays

The integrated form and extrachromosomal circular form of the ICEs in *S. suis* were detected by combination primers, P1–P4. The amplicons were sequenced by Sanger sequencing and aligned with the *attL* and *attR* of ICESsuHN105.

For mating experiments, the mutant strain P1/7 (Cm^R^, Tet^S^, and Ery^S^) was used as a recipient and the multi-drug resistant strain HN105 as the donor. The filter mating assays were performed as previously described (Huang J. et al., 2016;Huang K. et al., 2016). The cultured donor and recipient strains, at an OD_600_ of 0.4–0.6, were mixed at a ratio of 1:5. The cell mixtures were resuspended, spotted on filter membranes, and were grown on THB agar plates overnight. The transconjugants were selected using the antibiotics tetracycline/erythromycin and chloramphenicol. Finally, the transconjugants were further confirmed by PCR for the ICESsuHN105 genes *recN*, *gdh*, and *cps2*.

### Construction of *ΔSss* Mutant and Antimicrobial Susceptibility Test

The lantibiotic cluster deletion mutant strain Δ*Sss* (Cm^R^) was constructed as described above. For antimicrobial susceptibility testing, 5 μL of OD_600_ 0.6 *Streptococcus* culture was placed on the surface of THB agar (1.5%, w/v) plates and incubated at 37°C under 5% CO_2_ for 24 h. Melting agar (0.5% agar, w/v) medium was prepared and mixed with the indicator bacteria at about 50°C. The mixing agar medium (5 mL) containing 10^6^ CFU indicator bacteria was overlaid on the plate uniformly and then incubated at 37°C for 24 h. The antimicrobial susceptibility was measured by the appearance and diameter of the inhibition zone. The lantibiotic extraction was performed as previously described (Vaillancourt et al., 2015) and details are provided in the Supplementary Material.

## Results

### Characteristics of the Strain HN105

The bacterial strain isolated from a diseased pig with acute arthritis was identified as *S. suis* according to species-specific PCR (detecting *gdh* and *recN*) and 16S rRNA sequencing (Ishida et al., 2014), and named HN105. A remarkable self-agglutination resulted in a negative serologic agglutination test. Serotype-specific PCR identified HN105 as a type 5 strain of *S. suis* in genotype and showed α-hemolysis on 5% (v/v) sheep blood agar (Supplementary Figure S1). As HN105 was not serologically identified in the phenotype, we marked it as SS5 (genotype) here.

The *in vitro* growth characteristics of HN105 were compared with two other *S. suis* strains (P1/7 and ZY05719) in THB. The results indicated that the SS5 (genotype) strain HN105 grew faster at early logarithmic phase (Supplementary Figure S2).

### HN105 Exhibited High Virulence in Zebrafish and BABL/c Mouse Infection Models

**FIGURE 1:**
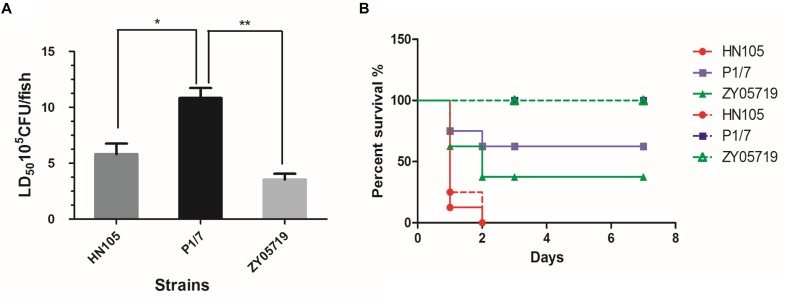
Animals experiments for strains HN105, P1/7 and ZY05719. The zebrafish **(A)** and BABL/c mouse **(B)** infection model were used to compare the virulence of SS5 (genotype) HN105 and SS2 P1/7, ZY05719. The dotted line means BABL/c mice with injection dose of 5^∗^10^7^ cfu. The solid line means BABL/c with injection dose of 2^∗^10^8^ cfu. The zebrafishes and BABL/c mice were observed 7 days after challenge. Statistical analyses were performed using one-way AVOVA and Tukey’s multiple comparisons test with all columns. *P* < 0.05 is labeled with ^∗^, *P* < 0.01 is labeled with ^∗∗^.

To evaluate the virulence of HN105 *in vivo*, we first used an established zebrafish model to compare its pathogenicity with two well-known SS2 virulent strains, P1/7 and ZY05719. The LD50 values for strains HN105, P1/7 and ZY05719 were 7.65 × 10^5^ CFU/fish, 1.08 × 10^6^ CFU/fish, and 3.52 × 10^5^ CFU/fish, respectively (Figure 1A). This result implied that the virulence of strain HN105 is close to that of ZY05719 and higher than that of P1/7 in the zebrafish model (Wu et al., 2014a).

Next, we used the BABL/c mouse infection model to reassess the virulence of HN105. As shown in the survival curves (Figure 1B), the HN105 strain was more pathogenic to BABL/c mice than the two SS2 strains, further supporting the idea that HN105 should be classified as part of the virulent group of *S. suis* (Dong et al., 2015). Furthermore, some of the mice infected with HN105 developed typical disease symptoms, including shivering and dyspnea.

### Antibiotic Resistance Profiles

The results of the MIC test were tabulated (Table 2). The data demonstrated that HN105 was resistant to many antibiotics such as tetracycline, kanamycin, gentamicin, erythromycin, lincomycin, neomycin, and spectinomycin. The *S. suis* strains P1/7 and ZY05719 were susceptible to these antibiotics, except for tetracycline, gentamicin and neomycin. This multidrug resistance, coupled with the high virulence of HN105 in animal infection assays, prompted a comprehensive genomics analysis of the SS5 (genotype) strain, HN105.

**Table 2:** The MIC of several antibiotics against *Streptococcus suis* HN105 and other two SS2 strains.

Antibiotic	HN105	P1/7	ZY05719	Resistance breakpoint

		MIC (μg/ml)		
Vancomycin	0.5	0.125	0.125	≥2
Tetracycline	128	32	32	≥8
Cephalosporins	<0.125	<0.125	<0.125	≥8
Chloramphenicol	8	1	2	≥16
Kanamycin	>256	8	16	–
Erythromycin	>256	<0.125	<0.125	≥1
Penicillin	<0.125	<0.125	<0.125	≥8
Lactobacillus	>256	>256	>256	–
Ampicillin	<0.125	<0.125	<0.125	≥8
Streptomycin	>256	4	>256	–
Doxycycline	32	0.25	0.25	≥16
Ciprofloxacin	64	8	32	≥4
Gentamicin	32	16	16	≥16
Amikacin	128	128	128	≥64
Norfloxacin	64	64	64	≥32
Lincomycin	>256	0.5	1	≥1
Neomycin	>256	128	128	–
Spectinomycin	>256	8	32	–


*“–” means there is no suitable breakpoint of *S. suis* for the AMR from CLSI.*

### General Features of the HN105 Genome

**FIGURE 2:**
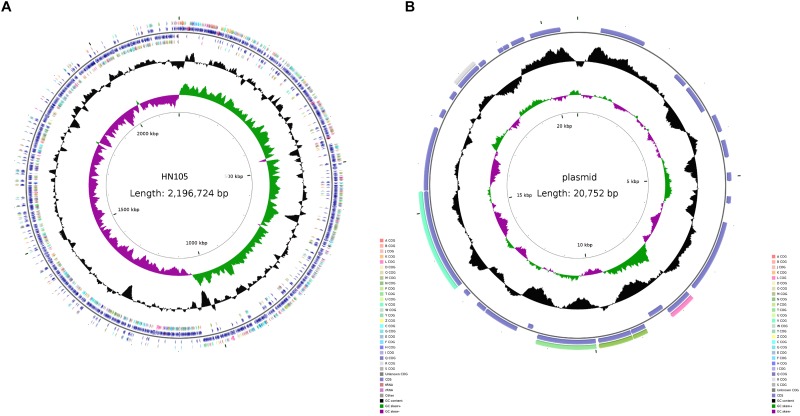
Circle map of the genome of *S. suis* HN105 **(A)** and its plasmid **(B)**. Map was established using the software CGView. The key information pertains to the circular diagrams (outside to inside): the outermost ring shows annotated CDSs, tRNA, rRNA, and GIs; the second ring shows GC content; the innermost ring shows GC skew [(G - C)/(G + C)].

The genomic sequencing of strain HN105 revealed a 2,131,882 bp circular chromosome and a 20,752 bp plasmid (Figure 2). The general features of the genome are summarized in Supplementary Table S2. In total, 2077 coding sequences (CDSs) were identified in the chromosome and 27 coding sequences on the plasmid. The average G+C contents were 41.4 and 33.22% for the chromosome and plasmid, respectively. Additionally, the tRNA and rRNA cluster copy numbers were similar to the other SS2 strains, and only one type of CRISPR was identified. MLST analysis identified that HN105 belongs to sequence type 498, which harbors another SS5 strain, Sdly140101.

### Comparative Genomic Analysis and Identification of a Novel ICE

**FIGURE 3:**
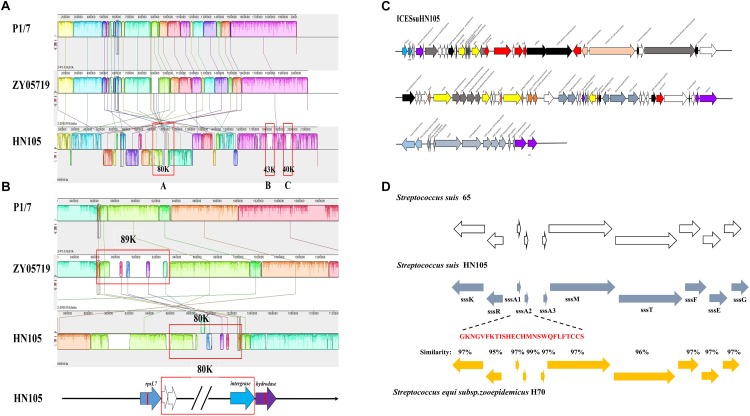
**(A)** Mauve comparison diagrams of the HN105, P1/7, and ZY05719 genomes. The map of an 80K genomic island, 43K genomic island and 40K genomic island were present in HN105 genome but absent in the virulent SS2 strains. **(B)** The enlarged map of 80K in HN105 and 89K in ZY05719. Each colored region is a locally collinear block (LCB). **(C)** A schematic map of the genetic structure of ICESsuHN105. The transcriptional directions and relative size of the ORFs are indicated by the arrows. The white arrows indicate unknown genes. The gray-blue arrows refer to the locus of lantibiotic cluster and the yellow arrows indicate the antibiotic resistant genes. The red arrows represent the T4SS system elements. The rest of the colored arrows are the essential genes for the complete function of ICEs. **(D)** An alignment between the type-B lantibiotic locus from *S. suis* strain 65 (white bars), *S. equi* subsp. H70 (yellow bars) and the corresponding locus within strain HN105 (blue bars) are shown. The nisin genes in *S. suis* are designated as *ssA1* and *ssA2*. The mature polypeptide is marked in red.

Comparative genomic analysis of HN105, the reference virulent SS2 strain P1/7 and ZY05719 was performed to better understand the multidrug resistance of the SS5 (genotype) strain. Genome alignment showed large-scale genomic rearrangements in HN105, including inversions, insertions and deletions. Although there was no typical 89K PAI encoded in HN105, a unique 80K genomic island (GI) was seen inserted in the chromosome. This GI was 79,639 bp in size with an average GC content of 38.04%, significantly different from that of the whole genome (41.4%). Genes encoding transposase and integrase were found in its flanking region. Although a lot of genes in 80K GI encode hypothetical proteins, we identified some antibiotic resistance-related genes or regulators, which will be interesting for further analysis (Figure 3A,B). In addition, there were two another GIs (43K and 40K) inserted into the chromosome, composed primarily of accessory secretion systems, glycosyltransferase and prophage elements.

Despite the large-scale genomic differences and the absence of important SS2-associated virulence genes such as *mrp* or *sly* in the chromosome of HN105, we identified some virulence-related factors such as *fbp*, *srtA*, *dippIV*, enolase, an orphan regulator, *covR* and the *nadR* regulator. In addition, the capsule synthesis gene *cps* and the capsule regulatory genes *ccpA* were also identified in HN105. Expanding the search to genes coding for products potentially involved in bacteremia-enhancing *S. suis* virulence revealed the presence of important virulence-related genes (Supplementary Table S3).

Further analysis of the 80K genomic island confirmed that it was a novel ICE element, possessing a conserved integrase closely related to intICESa2603 (>99% amino acid identity). Moreover, alignment of the core genes with ICESa2603 showed >60% DNA sequence identity. Both the above indexes are specific features of the ICESa2603 family^7^. Accordingly, the HN105 ICE was renamed ICESsuHN105 and classified as a member of the ICESa2603 family.

### Phylogenetic and Neighborhood Analysis of ICESsuHN105

In ICESsuHN105, integration between the *rpsL* and hydrolase genes yielded two 15 bp *att* sites at the left and right ends (*att*L/R: 5′-TTATTTAAGAGTAAC-3′). A putative *oriT* sequence was identified upstream of the *mobC* gene, at the genomic locus 1015147 to 1015183 (GGGATATTGTGGACACAATATCTGAGCTCGCAAAGAC), identical to ICESa2603. In addition to possessing genes involved in conjugation, which were annotated as a tyrosine family integrase, Tn5252 orf4 relaxase, Tn5252 orf9 *mobC* and Tn5252 orf10, ICESsuHN105 also contained some accessory genes conferring growth advantages (Figure 3C). Furthermore, ORFs predicted to encode the PezAT toxin–antitoxin system were verified to be functional and transcribed as an operon mode (Figure 3C).

As expected, the analysis results showed that ICESsuHN105 contains several antibiotic resistance-associated determinants. Examples include *HN015_04855* encoding an aminoglycoside *O*-phosphotransferase (APH), which could confer resistance to multiple antibiotics including kanamycin, lincomycin, neomycin, and amikacin; two identical genes (*HN015_04830* and *HN015_05090*) encode rRNA adenine *N*-6-methyltransferases for erythromycin resistance; genes *HN015_05015* and *HN015_04965* encode ribosomal protection proteins, which could protect the ribosome from the translation inhibition of tetracycline; and spectinomycin adenylyltransferase (*HN015_04990*) could result in spectinomycin resistance. Notably, a complete lantibiotic locus was encoded in this ICE (Figure 3D).

**FIGURE 4:**
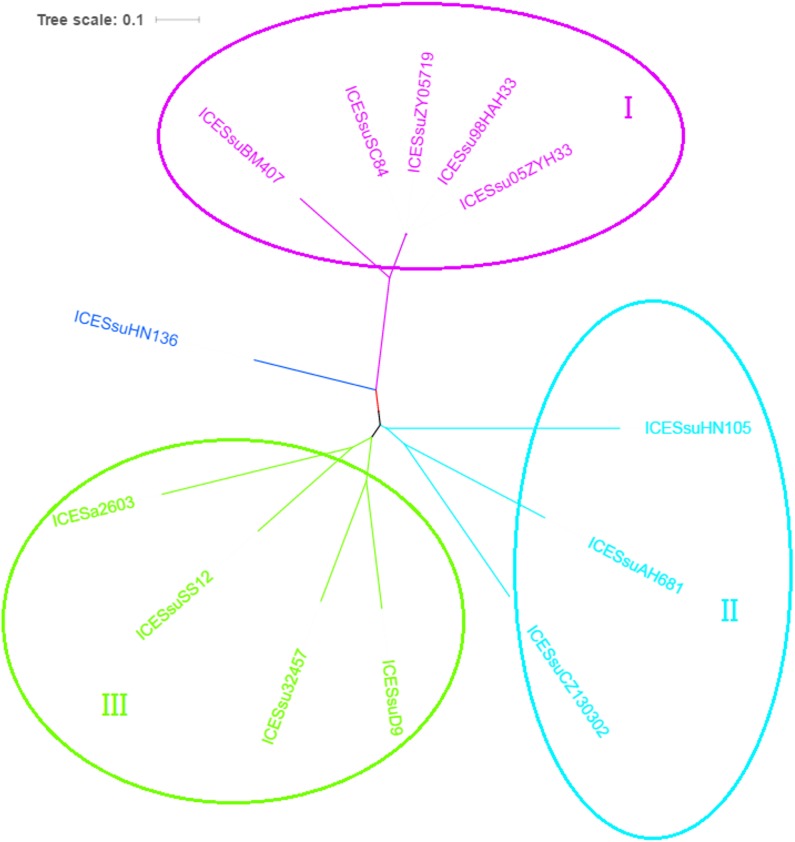
Phylogenetic tree of ICESa2603 family in *S. suis* based on backbone sequences built by concatenating core genes. The tree was constructed by maximum-likelihood method using MEGA6.

To analyze the evolution of the ICE core cluster in *S. suis*, we compared the DNA sequence identity of each of the ICE core genes to the corresponding genes of ICESa2603 (Figure 4). The phylogenetic trees based on the core genes demonstrated that the ICEs were further divided into three subgroups, with the divergent branch (group III) containing only one member, ICESsuHN136. Group I included 89K-like ICEs, most of them containing critical virulence-related factors, and tetracycline and macrolide resistance genes (Tang et al., 2006; Li et al., 2011). Three novel ICEs comprised group II, and included ICESsuHN105, ICESsuAH681 and ICESsuCZ130302. ICESsuCZ130302 and ICESsuAH681 were detected in novel serotype Chz strains. In addition, ICESsuCZ130302 has been demonstrated to be excisable from the donor cell chromosome, cyclized and transferred to the *S. suis* serotype 2 virulent reference strain P1/7 (Pan et al., 2019). These ICEs carried multiple antibiotic resistance genes instead of VFs.

### Effective Role of ICESsuHN105 in Resistance to Multiple Antibiotics

The resistance profiles of the wild-type and mutant Δ*80K* strains were compared for 7 antibiotics, to which HN105 and ZY05719 had been identified to be resistant and sensitive, respectively (Table 2). We found that the mutant strain was not resistant to any of these antibiotics (Table 3). Based on the data, we could preliminarily conclude that ICESsuHN105 is effective in conferring antibiotic resistance to HN105.

**Table 3:** Comparison of aminoglycosides antibiotics between *S. suis* HN105 and Δ*80K*.

Antibiotic	HN105	Δ*80K*

	MIC (μg/ml)	
Kanamycin	4096	32
Gentamicin	32	16
Neomycin	1024	128
Erythromycin	4096	<0.125
Tetracycline	128	16
Spectinomycin	4096	8
Lincomycin	1024	0.5


### Multidrug Resistance of ZY05719_(HN105)_ Results From the Insertion of Resistant Genes in Different Genomic Locations

The transmissibility of multidrug resistance from HN105 to the different *S. suis* strains was determined. Here we devised an experimental strategy to identify the genetic changes causing antibiotic resistance (Supplementary Figure S4). This strategy was involved to induce genomic hybrids by natural transformation between two *S. suis* strains with different phenotypic antibiotic sensitivity. Accordingly, purified HN105 genomic DNA was used as the donor DNA, with the SS2 ZY05719 being the recipient strain. The positive clones screened on THB plates containing multiple antibiotics were named ZY05719_(HN105)_ transformants. The antibiotic resistance of ZY05719_(HN105)_ was further investigated by MIC assays. The results indicated an increase in the MICs of spectinomycin, kanamycin, neomycin, tetracycline, and erythromycin for the ZY05719_(HN105)_ transformants compared to the parental strain (Table 4). This result indicated the antibiotic resistant genes could easily be horizontally transferred between clinical isolates from different serotypes under environmental antibiotic stress.

**Table 4:** The MIC results of ZY05719_HN105_ transformants.

Antibiotic	HN105	ZY05719_HN105_	ZY05719

	MIC (μg/ml)		
Kanamycin	>256	>256	32
Erythromycin	>256	>256	<0.125
Spectinomycin	>256	>256	32
Tetracycline	128	128	32
Neomycin	>256	>256	128


To explore whether horizontal transfer of antibiotic genes occurred by an insertion of the whole ICESsuHN105 box in the ZY05719 genome, one ZY05719_(HN105)_ transformant was sequenced and the draft genome was analyzed (GanBank ID: RZIC00000000). The genomic data suggested that the different antibiotic resistance elements from ICESsuHN105 were dispersedly inserted as single genes at different genomic locations. Although the acquisition of novel antibiotic resistance genes was confirmed by the contribution of the individual HN105-encoded genes, the experiments did not identify the horizontal transfer of intact ICESsuHN105 by natural transformation in *Streptococcus* species.

### Detection of Extrachromosomal Circular Forms of ICESsuHN105

**FIGURE 5:**
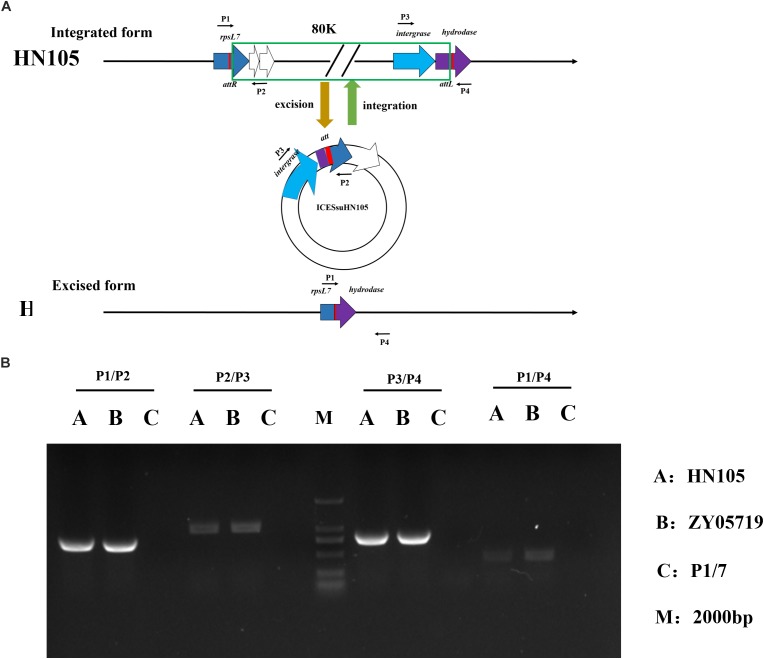
Circular form of integrative and conjugative element ICESsuHN015. **(A)** Diagrammatic representation of the integration and excision of ICESsuHN105 and the locations of detecting primers (shown by thin arrows) for the extrachromosomal form of ICESsD9 and genome walking assays. The left and right red rectangles represent the junctions (*attL* and *attR*), which can be formed by recombination. **(B)** DNA gel results of the PCR verifying the presence of the circular form of ICESsuHN105 and ICESsuZY05719.

Active ICEs can be excised from the chromosome with the aid of the integrase and excisionase to form circular extrachromosomal ICEs. Four specific primers (P1, P2, P3, and P4) targeting HN105 were designed in order to detect ICESsuHN105. PCR with different combinations of the four primers can be used to identify both the chromosomally integrated (P1–P2 and P3–P4 positive; P2–P3 and P4–P1 negative) and excised-circularized (P1–P2 and P3–P4 negative; P2–P3 and P4–P1 positive) forms of ICESsuHN105 in *S. suis* HN105 grown to logarithmic phase. The PCR analysis showed that DNA amplicons were obtained with all these four pairs of primers; however, the bands of P2–P3 and P4–P1 were shallower. The results indicated that although the probability of occurrence is relatively low, both the forms of ICESsuHN105 (chromosomally integrated, and excised-circularized) occurred when strain HN105 was cultured. This suggested that ICESsuHN105 had the ability to form an annular structure (Figure 5). However, successful conjugants between donor *S. suis* HN105 and recipient strains P1/7 (Cm^R^) have not been obtained in our *in vitro* conjugation assays.

### Identification of a Functional Lantibiotic Locus Located in ICESsuHN105

According to the comparative genomics analysis, there is a type B lantibiotic locus at the end of the 80K ICESsuHN105; an identical gene order to the nisin locus in *S. equi* subsp. strain H70 (Holden et al., 2009) and *S*. *suis* 65 (Vaillancourt et al., 2015). Based on the annotation, this complete lantibiotic locus (suicin locus) contains 10 coding genes, including a sensor histidine kinase (*sssK*), a response regulator (*sssR*), three precursors (*sssA1*, *sssA2*, and *sssA3*), a lantibiotic modification (*sssM*), an ABC transporter (*sssT*), and three immunity proteins (*sssF*, *sssE*, and *sssG)* (Figure 3D). Therefore, we redesignated this locus as ‘locus suicin 105.’

**FIGURE 6:**
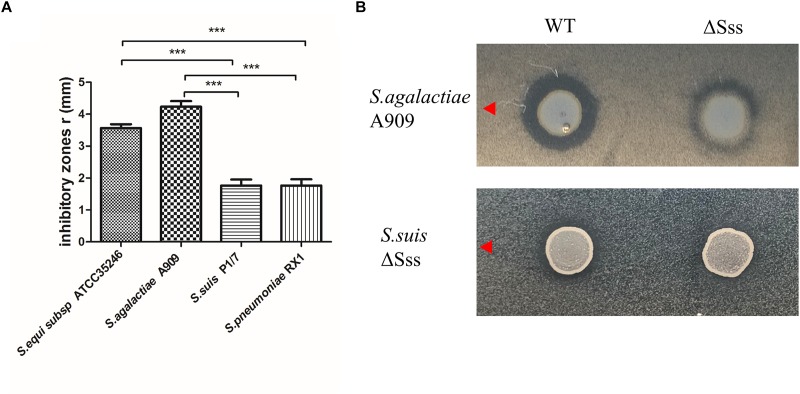
**(A)** Inhibitory zones produced by *S. suis* HN105 against 4 Streptococcus strains. **(B)** Inhibitory zones produced by *S. suis* HN105 and mutant (ΔSss) against *S. agalactiae* A909 and *S. suis*ΔSss. *P* < 0.001 is labeled with ^∗∗∗^.

The plate diffusion assay was used to investigate the antimicrobial ability of HN105 to other streptococcal strains. The data showed that *S. agalactiae* A909, *S. equi* subsp. *zooepidemicus* ATCC35246, *S. suis* P1/7, and *S. pneumoniae* RX1 were sensitive to HN105, but *S. agalactiae* GD1008-001 and *S. suis* ZY05719 were not (Figure 6A). Therefore, *S. agalactiae* A909 was selected as the indicator strain in further studies.

To verify that the observed antimicrobial ability of HN105 was conferred by suicin 105, a mutant strain ΔSss was constructed. Compared to the wild type, ΔSss displayed significantly smaller inhibitory zones in plate diffusion assays using *S. agalactiae* A909 as the indicator (Figure 6B). This result indicated that suicin 105 contributes to antimicrobial activity of HN105 against other streptococcal strains.

A previous study identified that bacteriocins were not secreted in a liquid medium (Vaillancourt et al., 2015). Accordingly, we purified suicin 105 after growth on THB plates using cationic exchange and reversed-phase MS, and a bacteriocin enrichment fraction (Thiede et al., 2014). Mass spectrometry identified the type of polypeptide that shared 92.3% identity with the predicted mature suicin peptide (Supplementary Table S4). This finding confirmed the secretion of suicin 105 in HN105.

## Discussion

Although no *S. suis* SS5 outbreak or epidemic has yet been reported, SS5 has been identified as a zoonotic pathogen (Kerdsin et al., 2011; Gustavsson and Rasmussen, 2014). In this study, the isolate HN105 was considered to be non-serotypeable with the reference antisera due to auto-agglutination. More and more isolates belong are non-serotypeable according to agglutination testing in clinic (Qiu et al., 2016; Zheng et al., 2017), which causes a problem for epidemiological investigation and genetic analysis. In this way, genotyping based on molecular techniques has been proposed based on serotype-specific *wzy*/*wzx* genes (Liu et al., 2013; Ishida et al., 2014; Okura et al., 2014; Athey et al., 2016), which may be a compromise to describe serotype in the genotype level. Some reports suggested that part of the non-serotypeable strains could be typed as the known serotypes by multiplex PCR, but could not be typed by the agglutination test (Zheng et al., 2015, 2017; Qiu et al., 2016). The reasons for phenotype of auto-agglutination, poly-agglutination or non-agglutination in strains with the reference antisera remain unclear until now (Zheng et al., 2015). Referring to the above studies, isolate HN105 could be grouped into serotype 5 according to capsular gene typing system. Here, the SS5 (genotype) strain HN105 presented a high virulence phenotype, similar to the representative virulent SS2 strains, in mouse and zebrafish infection models. A recent study identified a large number of virulence-related genes in *S. suis* that contribute to pathogenicity (Fittipaldi et al., 2012). BLAST analysis of the HN105 ORFs with the reference VFs identified 75.86% of the important virulence-related genes (23/31) (Supplementary Table S3). Despite the absence of several classical virulence-associated factors such as MRP, suilysin (Sly), EPF, and the orphan regulator RevS in HN105, some important VFs such as sortase A, dipeptidyl peptidase IV, Quorum sensing (LuxS), and other regulators (CiaRH, CovR, CcpA, and Rgg-like) are still conserved in the HN105 genome. For example, *ccpA* encodes an important global regulator and its deletion resulted in 259 differentially expressed genes as well as a markedly reduced capsule thickness that has a significant effect on virulence (Willenborg et al., 2011; Tang et al., 2012). Sortase A controls the capacity to adhere to and invade porcine brain microvascular endothelial cells (Vanier et al., 2008). In addition, some new VFs encoding putative transcriptional regulator or flagellar protein FliS were identified in the SS5 chromosome. Moreover, we also identified a response regulator LytR, a predicted plasmid-encoded virulence factor regulator (Virulence Factors of Pathogenic Bacteria database). Such examples emphasize the genomic diversity between different serotypes in *S. suis* (Dong et al., 2017; Zheng et al., 2018), which highlights the value of genomic information of non-SS2 strains. The mechanisms of virulence for the HN105 strain also need to be further investigated, such as the roles of ICESsuHN105, the 43K GI and 40K GI, and whether they contribute to animal infections in China, similar to ICESsuZY05719, the well-known 89K PAI, which remains unclear. We managed to test the contribution of this ICESsuHN105 in bacterial virulence with animal infection models, but it seems this 80K ICE was not required for the full virulence on HN105 isolate (Supplementary Figure S3). Thus, the following work attempts to explore its potential function on antibiotic resistance. Although 80K (ICE) did not influence virulence, the 40K and 43K GIs may play potential roles in the pathogenic process. In 43K, glycosyl transferase proteins and a Sec system were encoded. In 40K, a prophage was identified, which harbored some specific sections capable of opportunistic pathogenicity, including the proteolytic subunit ClpP, transcriptional regulator, holin and many hypothetical proteins. The similar structure of 50K and 58K GIs in SS serotype Chz strain CZ130302 (Zhang et al., 2018) had been determined play a key role in the pathogenic process. It indicated the role of these two GIs in the virulence of HN105.

Due to the widespread use of antibiotics in the porcine cultivation industry, *S. suis* is becoming increasingly resistant to numerous antibiotics (39). In this study, a novel ICESa2603 family member, ICESsuHN105, was identified in the pathogen SS5 (genotype) HN105. The strain was resistant to multiple antibiotics. Genome analysis revealed six antibiotic resistance genes in the HN105 genome that may confer resistance to diverse antibiotics by different protective pathways. For example, *tet(M)* and *tet(O)* were ribosomal protection proteins conferring tetracycline resistance. Specifically, the gene encodes aminoglycoside 3′-phosphotransferase (APH), which could catalyze the transfer of the gamma-phosphoryl group from ATP to aminoglycoside antibiotics such as kanamycin, streptomycin, neomycin and lividomycin (McKay et al., 1996). Phosphorylation of the aminoglycoside antibiotics results in their inactivation in a higher survival rate of bacterial cells (Yoon et al., 2014). BLAST analysis of this gene identified homologs in known multidrug resistant pathogens such as *Enterococcus faecalis, Staphylococcus aureus*, and *Campylobacter jejuni*. HN105 might also have acquired the *aph* gene through horizontal gene transfer because this gene is frequently found on transposons and plasmids, and is thought to have originated as a self-defense mechanism used by the microorganisms (Shi et al., 2013).

The similarity in the sequence identity of the integrase protein showed >99% homology between ICESa2603 and ICESsuHN105. Therefore, we grouped ICESsuHN105 into the ICESa2603 family. Indeed, the diversity of the phylogenetic tree patterns for the backbone genes of these ICEs indicated that frequent recombination events occurred in the process of ICE formation and evolution. These recombination events may cause ICESsuHN105 to adapt to the bacterial host and specific environments. Typically, the complete ICEs are thought to be capable of mobility and can propagate to other loci upon proper stimuli.

Interestingly, a complete suicin lantibiotic locus was encoded by ICESsuHN105. The bacteriocin production system in bacteria confers a growth advantage in the interbacterial antagonism. Numerous lantibiotics have been reported in *S. suis*, including suicin 90–1330, suicin 3908 and suicin 65 (LeBel et al., 2014; Vaillancourt et al., 2015). Suicin 105 shares 100% homology with suicin 65. This mature lantibiotic peptide has a globular portion in the C-terminal and a linear portion in the N-terminal. The mature suicin 105 also showed a high homology with nisin H70 (100%) and streptococcin A-FF22 (84.6%). The bacteriocin produced by these SS2 strains belonged to sequence type 28 (ST28), which is avirulent in animal models and can be treated as candidate vaccines (Athey et al., 2015). In contrast, the nisin locus found in a virulent SS5(genotype) strain HN105 genomic background could immunize and protect itself from bacteriocin killing, which may lead to failure for lantibiotic bacteriocin with potential prophylactic applications (Bierbaum and Sahl, 2009). One can easily envision a scenario where the mobile ICE with bacteriocin could integrate into other microorganisms, including other pathogens that we want to target. Such a multidrug-resistant pathogenic strain with remarkable competitive advantage for interbacterial antagonism in the environment could be a greater risk for human health.

Despite the failed *in vitro* conjugation assay, our DNA hybridization experiment by natural transformation resulted in the transfer of antibiotic resistance genes into different isolated genomic backgrounds. The capability of natural transformation is an important mechanism for evolution and chromosome diversity in *Streptococcus* species (Antunes et al., 2010). The active ComRS competence system found in the *S. suis* genome provides this strain with the ability to non-specifically uptake foreign genetic material, including genes coding for antibiotic resistance and virulence factors.

## Conclusion

The data indicates that the SS5 (genotype) strain HN105 may be a potential source for the spread of antibiotic resistance genes and may cause serious problems regarding drug resistance.

## Data Availability

The datasets generated for this study can be found in NCBI, The access number of the complete genome sequence for strain HN105 is CP029398.

## Ethics Statement

The BABL/c mice were purchased from the Comparative Medicine Center of Yangzhou University. Zebrafish were purchased from the Pearl River Fishery Research Institute, Chinese Academy of Fishery Science. Animal experiments were performed in the Laboratory Animal Center of Nanjing Agricultural University with the approval of the Laboratory Animal Monitoring Committee of Jiangsu Province, China (Permit number: SYXK (Su) 2017-0007). All animal experiments complied with the guidelines of the Animal Welfare Council of China.

## Author Contributions

YCZ, YZ, and WD conceived and designed the experiments. YCZ performed the experiments and analyzed the data. YCZ, WD, and XZ prepared the draft paper. ZP and JM supervised the laboratory work. JM, ZP, and HY reviewed and edited the manuscript. All authors approved the final manuscript.

## Conflict of Interest Statement

The authors declare that the research was conducted in the absence of any commercial or financial relationships that could be construed as a potential conflict of interest.

## Footnotes

1 http://www.pathogenomics.sfu.ca/islandviewer/upload/

2 http://www.ncbi.nlm.nih.gov/genbank/

3 http://ssuis.mlst.net/

4 http://eburst.mlst.net/

5 https://www.ncbi.nlm.nih.gov/

6 http://blast.ncbi.nlm.nih.gov/Blast.cgi

7 http://db-mml.sjtu.edu.cn/ICEberg/index.php
