# Supplementary material for: ICESsuHN105, a Novel Multiple Antibiotic Resistant ICE in Streptococcus suis Serotype 5 Strain HN105
Source: Front Microbiol. 2019 Feb 26;10:274. doi: 10.3389/fmicb.2019.00274 (PMC6399138; doi:10.3389/fmicb.2019.00274)
Supplement: Supplementary file 1 [file Data_Sheet_1.PDF]

SUPPLEMENTARY MATERIAL FOR

ICESsuHN105, a novel multiple antibiotic resistant ICE in *Streptococcus suis* serotype 5 strain HN105

Yinchu Zhu[1,2,3], Yue Zhang[1,2,3], Jiale Ma[1,2,3], Wenyang Dong[1,2,3], Xiaojun Zhong[1,2,3], Zihao Pan[1,2,3], Huochun Yao[1,2,3§]

[1]College of Veterinary Medicine, Nanjing Agricultural University, Nanjing, China

[2]OIE Reference Lab for Swine *Streptococcosis*, Nanjing 210095, China

[3]Key Lab of Animal Bacteriology, Ministry of Agriculture, Nanjing Agricultural University, Nanjing 210095, China

[§]Corresponding author: Huochun Yao

Email: yaohch@njau.edu.cn

Tel: +8625 84395328

Fax: +8625 84395328

Materials and Methods

Extraction and identification of *S. suis* lantibiotic

The lantibiotic extraction was referred to a previously described method (Vaillancourt et al., 2015a). One hundred μl HN105 culture was spread onto THB agar plates supplemented with 0.25% glucose and 0.01% Tween 80, incubation at 37°C under CO_2 conditions for 24 h. Bacterial were removed and the agar of plate was chopped into small pieces and transferred 2 ml 20 mM 2-(N-morpholino) ethanesulfonic acid (MES) buffer (pH 5.5, 0.01% Tween 80). The mixture was placed at -80°C overnight, and then thawed at 55°C After a high centrifugation over 13,000 ×*g*, the supernatant was collected. Above procedures need to be repeated once more to remove remaining bacteria and agar particles in bacteriocin fraction. Ammonium sulfate solid was slowly added to the bacteriocin fraction to final concentration of 70% (w/v), and the mixture was stirred for 3h at 4°C, then the supernatant was centrifuged with 13,000 ×*g* for 15 min at 4°C, discard the supernatant. Finally, the precipitate was suspended in 1 x PBS (pH 7.2).

The crude extract was dialyzed against a 1KD RC dialysis cartridge, 20 mM MES (0.01% Tween 80, pH 5.5) was used. It was separated and purified by Sephacry1S-300 gel column (NGC chromatography system) after being dialyzed at 4 ℃ for 48 h. The MES as mobile phase with the flow rate was 0.3 mL / min and the detection wavelength was 215 nm. After the determination of antibacterial activity, samples were sent for protein sequencing using mass spectrometry (MS) system.

Results

Table S1. Primes used in the follow studies

Name	Sequence(5'-3')	Size(bp)	Functional
Cps5I-F	TTTTCGTTGTATTTTCCAAA	262	Serotype 5 gene of *S.suis*
Cps5I-R	TCCAAACATTATCCCCTATT		
recN-F	CTACAAACAGCTCTCTTCT	336	Identification for *S.suis*
recN-R	ACAACAGCCAATTCATGGCGTGATT		
gdh-F	CCATGGACAGATAAAGATGG	688	Identification for *S.suis*
gdh-R	GCAGCGTATTCTGTCAAACG		
16sRNA-F	AGAGTTTGATCTTGGCTCAG	1465	Sequencing primers
16sRNA-R	TACGGGTACCTTACGACTT		
80K-1	CTGGTCAAAAAGTCCAAAGTC	1371	Upper flanking for 80KGI deletion
80K-2	TTCAGCATTATCCTGAAGCGCCTGCTTCTTCCAA		
80K-3	ACCCATCGAATTATAGAGTTTCCAATCAAAATA	1169	Reverse flanking for 80KGI deletion
80K-4	CTCGTAGAGTTTGACAGGAAT		
80K-5	AACTGACCTAAGACAGTAGGG	4873	Fusion PCR for Homologous arm
80K-6	ATCTGATAAGCCGCTTCTGAG		
P-1	AAAGTTGGCGTTATCAAAG	728	P1/P2 for excision of ICE
P-2	GCCCCATCCTCATCAATCC	1038	P2/P3 for excision of ICE
P-3	AACAAAGACTCCATCAGGTGA	700	P3/P4 for excision of ICE
P-4	GCGGTCGATAGGAACAACC	428	P1/P4 for excision of ICE
CAT-F	CACCGAACTAGAGCTTGATG	1056	Chloramphenicol gene
CAT-R	TAATTCGATGGGTTCCGAGG		
cpn60-F	TTGAAAAACGTRACKGCAGGTGC	318	cpn60 gene for MLST
cpn60-R	ACGTTGAAIGTACCAAGAATC		
MutS-F	ACTGGGGCATAAGGTCG	339	MutS gene for MLST
MutS-R	TGGCTGTCTTAGTTTCGTC		
thrA-F	GATTCAGAACGTCGCTTTGT	336	thrA gene for MLST
thrA-R	AAGTTTTCATAGAGGTCAGC		
aroA-F	TTCCATGTGCTTGAGTCGCTA	336	aroA gene for MLST
aroA-R	ACGTGACCTACCTCCGTTGAC		
dpr-F	CGTCTTTCAGCCCGCGTCCA	336	dpr gene for MLST
dpr-R	GACCAAGTTCTGCCTGCAGC		
gki-F	GGAGCCTATAACCTCAACTGG	321	gki gene for MLST
gki-R	AAGAACGATGTAGGCAGGATT		
RecA-F	TATGATGAGTCAGGCCATG	354	RecA gene for MLST
RecA-R	CGCTTAGCATTTTCAGAACC		
Sss-1	AGAGATTGAAAATCGATTAG	1192	Upper flanking for gene deletion
Sss2	TCTAGTTCGGTGAGATATTTTTGAGGATACCTT		
Sss-3	ACCCATCGAATTATTTTAAATAAATCGCTTCTAT	1291	Reverse flanking for gene deletion
Sss-4	CTAGGAGTTATTGCTTTTAAT		
Sss-5	ACTTTAAAAATAAGGAAGTAGGTG	3101	Fusion PCR for Homologous arm
Sss-6	TAGCTTTCTTTTGTACCCTT		

Table S2 General properties of the genome of strain HN105

Replicon	Size(bp)	GC%	CDS	rRNA	tRNA	CRISPRS
Chr	2,196,724	41.40	2077	12	56	1
plasmid	20,752	34.22	27	0	0	0

Table S3 Comparison of predicted virulence-associated factors in HN105, P1/7, and ZY05719

Gene description		Strains		
		HN105	P1/7	ZY05719
**Proteinase**				
	Glutamate dehydrogenase (GDH)	+	+	+
	Sortase A	+	+	+
	Extracellular protein factor (EPF)	-	+	+
**Adhesin**				
	Fibronectin binding protein (FBP)	+	+	+
	Muramidase-released protein (MRP)	-	+	+
	Glyceraldehyde-3-phosphate dehydrogenase	+	+	+
	Glutamine synthetase	+	+	+
	Dipeptidylpeptidase IV	+	+	+
	Hemagglutinin	-	+	+
	Enolase	+	+	+
	6-phosphogluconate dehydrogenase	+	+	+
**Hemolysin**				
	Suilysin (SLY)	-	+	+
**TCSTS**				
	CiaRH	+	+	+
	Orphan response regulator(CovR)	+	+	+
	Orphan response regulator(RevS)	-	+	+
	VraRS	+	+	+
	SalK/R	-	+	+
**Regulator**				
	Zinc uptake regulator	+	+	+
	Gene homologous to *S. mutans* SMU_61	+	+	+
	Iron uptake regulator	+	+	+
	Sugar catabolism regulator(CcpA)	+	+	+
	Transcriptional regulator(nadR gene)	-	+	+
	Transcriptional regulator(Rgg-like)	-	+	+
	putative transcriptional regulator	+	-	-
**Others**				
	Resistance to zinc-mediated toxicity	+	+	+
	Quorum sensing(LuxS)	+	+	+
	Resistance to acidity(arcA, arcB, arcC)	+	+	+
	LTA D-alanylation(DltA)	+	+	+
	Subtilisin-like protease(SspA)	+	+	+
	Degradation of host surface oligosaccharides	+	+	+
	Degradation of host DNA(SsnA)	+	+	+
	Manganese uptake(TroA)	+	+	+
	ABC transporter ATPase	+	-	-
	putative flagellar protein FliS	+	-	-

Table S4 The polypeptide identified by mass spectrum analysis

Sequence	Coverage	Charge	MH+	Engine
GKNGVFKTISHECHMNSWQFLFTC	92.5%	3	2560	MASCOT

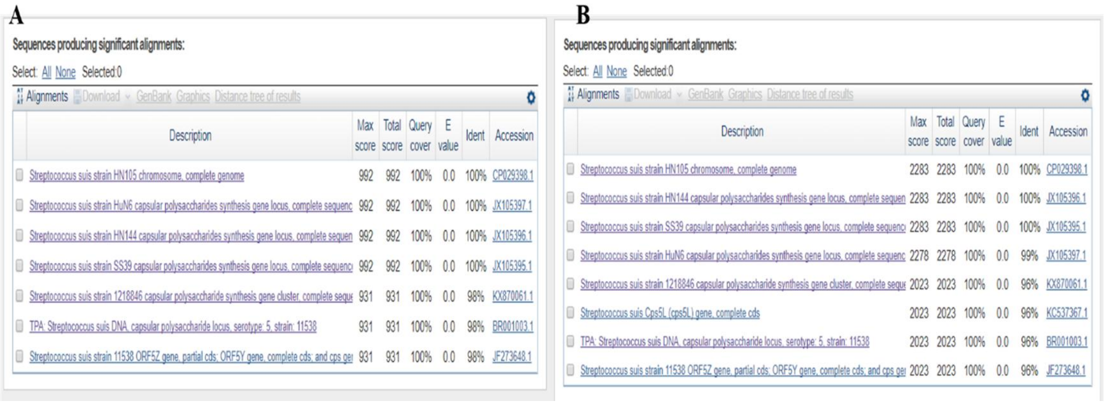
A

Sequences producing significant alignments:

Select: All None Selected:0

Alignments Download GenBank Graphics Distance tree of results

Description	Max score	Total score	Query cover	E value	Ident	Accession
Streptococcus suis strain HN105 chromosome, complete genome	992	992	100%	0.0	100%	CP029398.1
Streptococcus suis strain HuN6 capsular polysaccharides synthesis gene locus, complete sequenc	992	992	100%	0.0	100%	JX105397.1
Streptococcus suis strain HN144 capsular polysaccharides synthesis gene locus, complete sequen	992	992	100%	0.0	100%	JX105396.1
Streptococcus suis strain SS39 capsular polysaccharides synthesis gene locus, complete sequenc	992	992	100%	0.0	100%	JX105395.1
Streptococcus suis strain 1218846 capsular polysaccharide synthesis gene cluster, complete seque	931	931	100%	0.0	98%	KX870061.1
TPA: Streptococcus suis DNA, capsular polysaccharide locus, serotype: 5, strain: 11538	931	931	100%	0.0	98%	BR001003.1
Streptococcus suis strain 11538 ORF5Z gene, partial cds; ORF5Y gene, complete cds; and cps ger	931	931	100%	0.0	98%	JF273648.1

B

Sequences producing significant alignments:

Select: All None Selected:0

Alignments Download GenBank Graphics Distance tree of results

Description	Max score	Total score	Query cover	E value	Ident	Accession
Streptococcus suis strain HN105 chromosome, complete genome	2283	2283	100%	0.0	100%	CP029398.1
Streptococcus suis strain HN144 capsular polysaccharides synthesis gene locus, complete sequen	2283	2283	100%	0.0	100%	JX105396.1
Streptococcus suis strain SS39 capsular polysaccharides synthesis gene locus, complete sequenc	2283	2283	100%	0.0	100%	JX105395.1
Streptococcus suis strain HuN6 capsular polysaccharides synthesis gene locus, complete sequenc	2278	2278	100%	0.0	99%	JX105397.1
Streptococcus suis strain 1218846 capsular polysaccharide synthesis gene cluster, complete seque	2023	2023	100%	0.0	96%	KX870061.1
Streptococcus suis Cps5L (cps5L) gene, complete cds	2023	2023	100%	0.0	96%	KC537367.1
TPA: Streptococcus suis DNA, capsular polysaccharide locus, serotype: 5, strain: 11538	2023	2023	100%	0.0	96%	BR001003.1
Streptococcus suis strain 11538 ORF5Z gene, partial cds; ORF5Y gene, complete cds; and cps ger	2023	2023	100%	0.0	96%	JF273648.1

Fig. S1 The sequence identifies blast of *wzx* and *wzy* genes in *S. suis*. (A) *wzx* gene show different identifies between SS5 strains (>96%). (B) *wzy* gene show different identifies between SS5 strains (>98%).

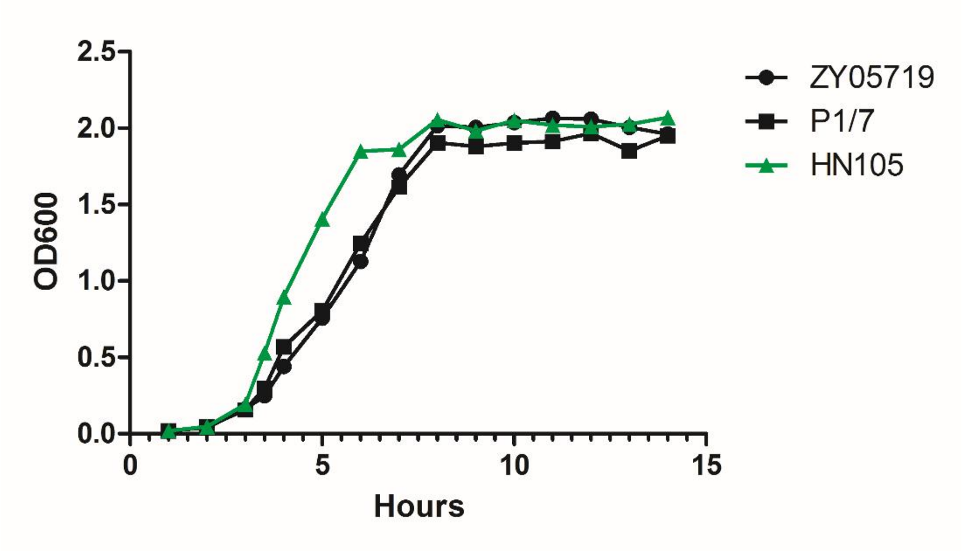

Fig. S2 The growth curve of strains HN105, P1/7 and ZY05719 using OD_{600} values.

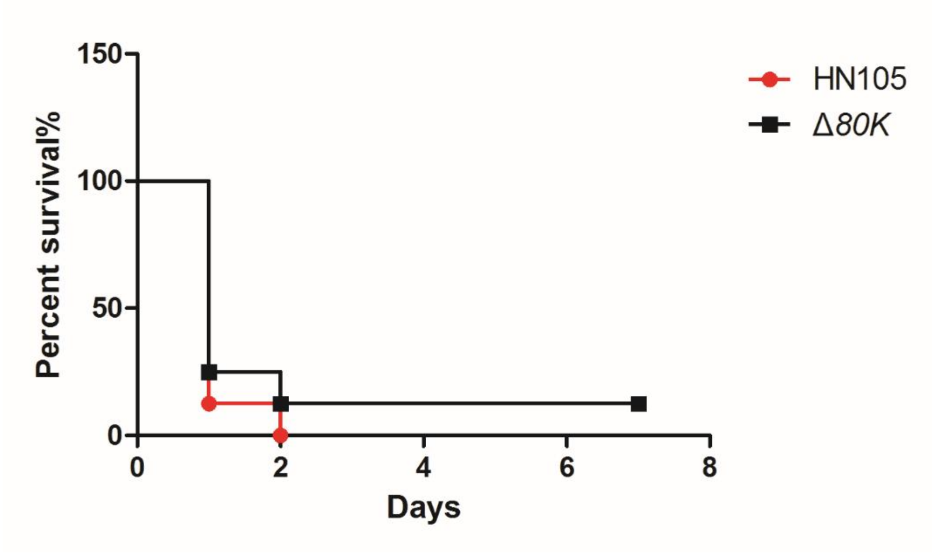

Fig. S3 The survive curve of HN105 and Δ*80K* with 5*10^7CFU on BABL/c mice.

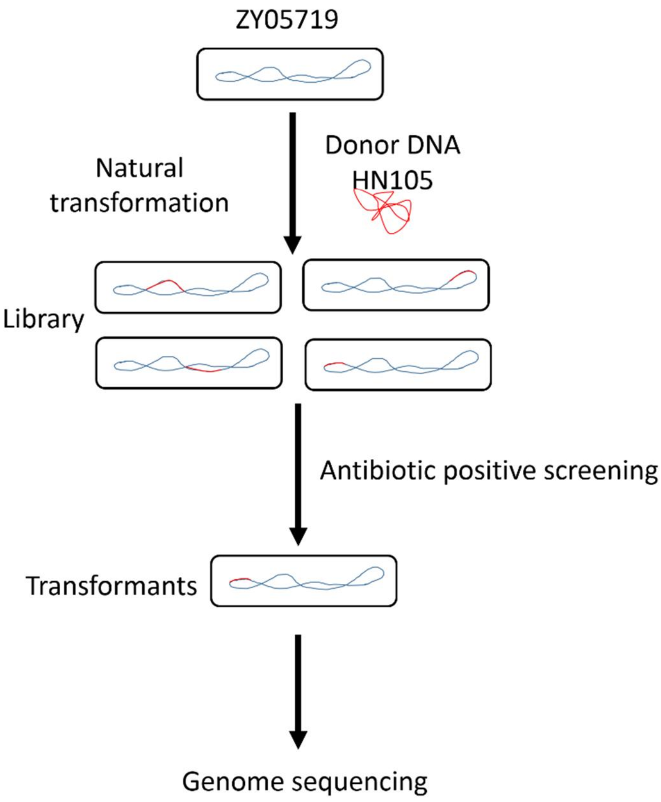

Fig. S4 The strategy of bacteria DNA hybridized applied in this study. The DNA of multi-antibiotic isolate HN105 as the donor template and transferred into ZY05719. Identification of these swapped genes by genome sequencing.
